# *In Silico* Target-Specific siRNA Design Based on Domain Transfer in Heterogeneous Data

**DOI:** 10.1371/journal.pone.0050697

**Published:** 2012-12-21

**Authors:** Qi Liu, Han Zhou, Kui Zhang, Xiaoxiao Shi, Wei Fan, Ruixin Zhu, Philip S. Yu, Zhiwei Cao

**Affiliations:** 1 Department of Bioinformatics, Tongji University, Shanghai, China; 2 Department of Computer Science, University of Illinois at Chicago, Chicago, Illinois, United States of America; 3 IBM Thomas J. Watson Research Center, New York, New York, United States of America; St. Georges University of London, United Kingdom

## Abstract

RNA interference via exogenous small interference RNAs (siRNA) is a powerful tool in gene function study and disease treatment. Designing efficient and specific siRNA on target gene remains the key issue in RNAi. Although various *in silico* models have been proposed for rational siRNA design, most of them focus on the efficiencies of selected siRNAs, while limited effort has been made to improve their specificities targeted on specific mRNAs, which is related to reducing off-target effects (OTEs) in RNAi. In our study, we propose for the first time that the enhancement of target specificity of siRNA design can be achieved computationally by domain transfer in heterogeneous data sources from different siRNA targets. A transfer learning based method *i.e.*, heterogeneous regression (HEGS) is presented for target-specific siRNA efficacy modeling and feature selection. Based on the model, (1) the target regression model can be built by extracting information from related data in other targets/experiments, thus increasing the target specificity in siRNA design with the help of information from siRNAs binding to other homologous genes, and (2) the potential features correlated to the current siRNA design can be identified even when there is lack of experimental validated siRNA affinity data on this target. In summary, our findings present useful instructions for a better target-specific siRNA design, with potential applications in genome-wide high-throughput screening of effective siRNA, and will provide further insights on the mechanism of RNAi.

## Introduction

RNA interference (RNAi) is a post-transcriptional gene silencing process during which the expression of endogenous mRNA is blocked by introducing a double-strand RNA (dsRNA) [1]. The mechanism of RNAi can be summarized as following: First, the Dicer enzyme binds dsRNA and cleaves it into 21∼23 nt fragments named short interference RNA (siRNA). Then, the siRNA loads onto the RNA-induced silencing complex (RISC) and separates into the guide strand (antisense to the target mRNA) staying in the RISC, and the passenger strand (sense to the target mRNA) released and degraded [2]. Next, the siRNA-RISC complex recognizes the target mRNA with guide strand pairing up with the complementary mRNA sequence [3]. Finally, the target mRNA is cut by Ago protein, leading to an efficient inhibition of gene expression.

RNAi is a simple, effective and low-cost technology which is of extensive applications such as gene function investigation, drug target discovery and disease treatment [4]. In a notably short time since their development, siRNAs have entered human clinical trials in various disease areas [5], [6], [7]. However, the silencing ability of different siRNAs varies widely. Rapid acceptance of the use of siRNAs has been accompanied by recognition of several obstacles for RNAi technology, such as the lack of specificity to a target mRNA, commonly termed as the “off-target effects” (OTEs) [8]. OTEs can complicate the interpretation of phenotypic effects in gene-silencing experiments and potentially lead to unwanted toxicities [5]. Hence, the efficient and specific design for siRNAs has become two important issues in RNAi. Here, efficiency means that a siRNA inhibits the expression of target mRNA exhaustively, while specificity means that a siRNA would better not affect the non-target mRNAs as to avoid an “off-target” effect. These two principals have been concerned and followed by a number of research groups in their studies, during which a series of criteria for siRNA design are addressed [9], [10], [11], [12]. Among them, a number of the criteria are generally proposed to improve the efficiency in siRNA design from different perspectives, *i.e.* (1) siRNA sequence features; (2) siRNA sequence motifs; (3) thermodynamic features of siRNA duplexes and their targets; and (4) other structural features for optimal siRNA-mRNA interactions [13], [14]. Besides the above criteria concerning about efficiency, there are several ways to further improve the specificity and reduce OTEs in siRNA design. For example, a “siRNA pool” technology is used to silence mRNA with lower concentrations of a set of siRNAs [15], and chemical modification like the locked nucleic acids (LNA) modification [16] is introduced into siRNA at both sequence level and structure level to improve their specificity.

With the increasing siRNA data, various statistical machine learning methods have been developed for siRNA efficacy analysis to derive more specific in-silico siRNA design rules. This procedure is generally formulated in a training-testing phase. The training data consist of a collection of siRNA sequences with related inhibiting affinity vis-a-vis their target genes, and these data are used to train a siRNA affinity prediction model. In the testing phase, trained models are applied to new instances to predict siRNAs affinity and perform feature selection. In 2005, Novartis published a relatively large siRNA dataset and modeled their affinity with neural network [17]. Following this work, Shabalina et al. [10] performed further thermodynamic and correlation analyses. Using four independent databases, Matveeva et al. [18] developed a new method for predicting siRNA efficacy with linear regression. In 2009, Klingelhoefer et al. [19] applied a Bayesian analysis on several combined datasets to identify a number of features associated with siRNA affinity. Nevertheless, although these studies have made considerable progress in siRNA design, we found that there still exist significant issues to be addressed, which are listed below.

### 1. Improper integration of the cross-platform siRNA data

A number of RNAi datasets are publicly available but each dataset was typically generated by a different group possibly using a different platform under specific experimental conditions (*i.e.* different cell types, test methods, siRNA delivery methods and siRNA concentrations), making integrated analysis and utilization of these datasets a challenge [20]. Different studies might use different measurements to assess the siRNA efficacy, thus leading to the different data distribution, *i.e.*, heterogeneity among different datasets. We observed from our previous study that generally the siRNA efficacy for different platforms cannot be easily compared, hence making a simple-mind integration of heterogeneous datasets hardly useful [20], [21]. For a given siRNA dataset, how to make use of data from other related datasets becomes a critical issue, since the number of samples from individual dataset is small and the design rules derived from such individual dataset may not be statistically significant. This issue can be further explained by that the empirical rules obtained through studies on individual dataset have been questioned about their general applicability [14], [19], [21], [22], [23]. And the rules proposed by different groups may be inconsistent. For instance, Saetrom et al. claimed that the sequence information alone can determine the efficacy of siRNAs [23] while several other groups suggested that thermodynamic features are important to siRNAs effectiveness. In our previous study [21], we have applied an aggregated ranking method to derive the common features for siRNA design across different heterogonous datasets.

### 2. Inadequate consideration of the specificity of target mRNAs

It has been proposed in the recent studies that the effect of siRNAs on individual gene is not only influenced by site-specific factors, such as the sequence match between siRNA-mRNA and the local RNA structure, but also depend on other system-level factors like the target mRNA cellular abundance and turn-over rate [24], [25] in the whole cell environment. Considering the properties of mRNA do play an important role in determining the binding efficacy of a siRNA, we believe that the general siRNA design guidelines derived from individual dataset need to be re-considered and improved. We propose that the traditional in-silico models for siRNA efficacy prediction may no longer work properly for the large-scale cross-platform data and more concerns on the specificity of different target genes should be paid [20], [21]. It should be noted that when such specificity is concerned, two points are needed to be raised firstly, (1) Since the system-level factors of mRNA may influence the siRNA efficacy, we conjecture that siRNA affinity prediction model should be built based on a “granularity” of mRNA, that it, it is better to compile siRNA affinity data targeted on the same mRNA to build the prediction model, rather than traditional work to mix the data from different targets, since affinity of siRNA targeting on other mRNAs may bias the design on the current target. (2) When we treat each mRNA and its existing binding siRNA as an independent dataset, the general problem faced here is that commonly the number of experimental RNAi data toward a specific target mRNA is small, and they are insufficient to train an accurate predictive model or derive statistical significant design rules for more efficient and specific siRNAs design.

These two points seem to be contradictory to each other. On the one side, we need to consider siRNA dataset in a granularity of mRNA. On the other side, the number of existing siRNA data with measured affinity on a specific target mRNA is generally insufficient. Nevertheless, if we treat the siRNA data on each specific target as different task or domain, a probably solution to “unify” them is to perform “knowledge transfer” across the domain/task, which helps to solve the data sparseness within a specific domain/task.

In summary, two basic problems are raised and studied in our paper: (1) if the existing siRNA affinity data for a specific mRNA (Target mRNA) is insufficient, can we leverage the information on other targets (Source mRNA) to help to construct the computational model for current target? That is, can we “transfer” the knowledge from source mRNA to target mRNA to help its siRNA design? (2) how to select suitable source mRNAs for knowledge transfer, and what is the influence of different source mRNAs on the target mRNA [26]?

In order to addressing these two problems, we formulate the target-specific siRNA design in a transfer learning-based schema, *i.e.*, heterogeneous regression (HEGS) [27]. Our extensive analysis indicate that HEGS can help to build a more accurate model for target-specific siRNA efficacy prediction, alleviating the insufficient of training data taking the advantages of data transferred from homogeneous genes, and substantially improving the design for siRNAs with more affinity specific to the given target mRNAs.

## Materials and Methods

### Dataset

Ten experimental validated siRNA efficacy datasets from different research groups were used in our study [19]. The datasets were limited to siRNA sequences targeted at mammalian mRNAs. By convention, siRNA sequences were represented as anti-sense sequences from 5′ to 3′ and the siRNA potency was measured by the mRNA/protein product levels after gene silencing. A detailed description of the ten datasets is presented in our previous published work [21]. Note that the siRecord data (Ren, et al., 2006) was excluded from our study since the data used categorical values, unlike continuous values used in the other datasets in measuring the siRNA potency. The remaining datasets contain nearly all the RNAi data using numerical siRNA efficacy values reported so far. In totally 4,482 unique and experimentally validated 19 nt siRNAs along with their efficacy values. The same 497 features proposed by Klingelhoefer et al. [19] were adopted as the starting point of our study, including compositional, thermodynamic and structural features, which is believed to be a comprehensive feature description for siRNA. These ten datasets were treated as “cross-platform”, with heterogeneity from each other.

Besides these ten cross-platform datasets, 31 sub-datasets compiled from the largest dataset, *i.e.*, Novartis's dataset, were also used in our study. The 31 sub-datasets were divided based on the “granularity” of mRNA. That is, the siRNA samples targeted on the same mRNA were just taken as a sub-dataset, totally 31 sub-datasets targeted on 31 mRNAs (Table 1). Such data was used to test our model on the target-specific siRNA design.

**Table 1:** Description for 31 siRNA sub-datasets.

Sub-dataset ID	mRNA with GenBank accession number	Data Size
1	BD135193	67
2	NM_004359	57
3	NM_003340	78
4	NM_003337	79
5	NM_003969	76
6	XM_371822	145
7	NM_015213	126
8	NM_003347	53
9	NM_003344	70
10	NM_003345	64
11	NM_014501	79
12	NM_004223	72
13	NM_016406	70
14	NM_014176	77
15	NM_001001481	78
16	NM_016021	49
17	NM_005339	79
18	NM_053656	77
19	NM_003348	79
20	NM_003342	79
21	NM_006357	79
22	NM_021988	74
23	NM_022005	72
24	XM_214061	144
25	NM_017346	46
26	NM_012864.1	75
27	NM_012864.2	75
28	NM_025237	75
29	NM_005450	71
30	NM_007019	76
31	NM_002559	90

### General workflow of our experiment design

**Figure 1:**
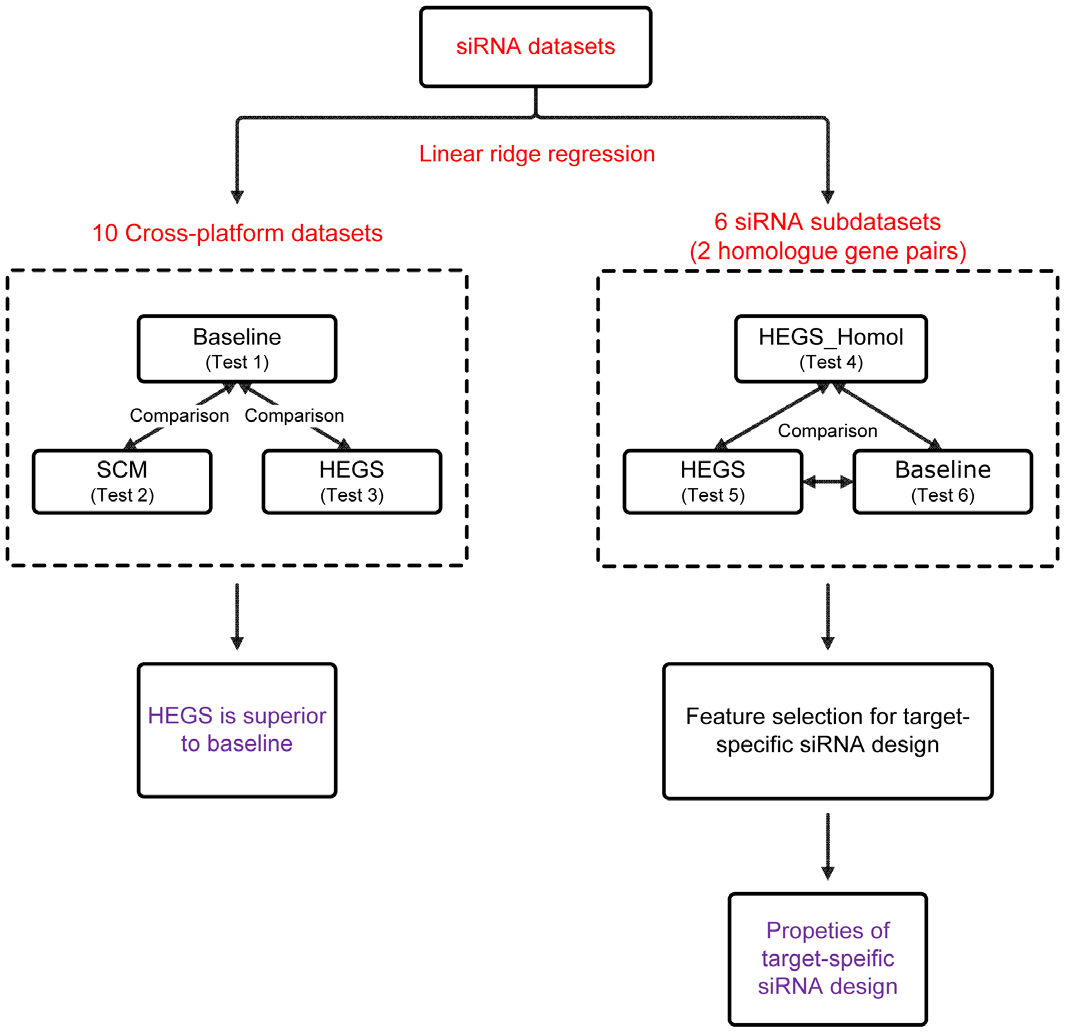
Computational framework of our study.

The computational framework of our study is presented in Figure 1, which can be divided into two parts: (1) The first part was performed based on the 10 cross-platform siRNA datasets. The simple linear regression model (baseline), was compared with a simple data combination and normalization method (termed as simply-combined model, SCM) as well as our proposed domain transfer based model (HEGS) [27], in terms of the model prediction performance (Tests 1–3). (2) The second part was performed based on the 31 target-specific siRNA sub-datasets. How different domains of source data influence the result of HEGS model was investigated, and then, a couple of homologous genes were selected as an example to demonstrate our feature selection for target-specific siRNA design (Tests 4–6). A detailed description of each test is listed in File S1.

### General outline for HEGS model

**Figure 2:**
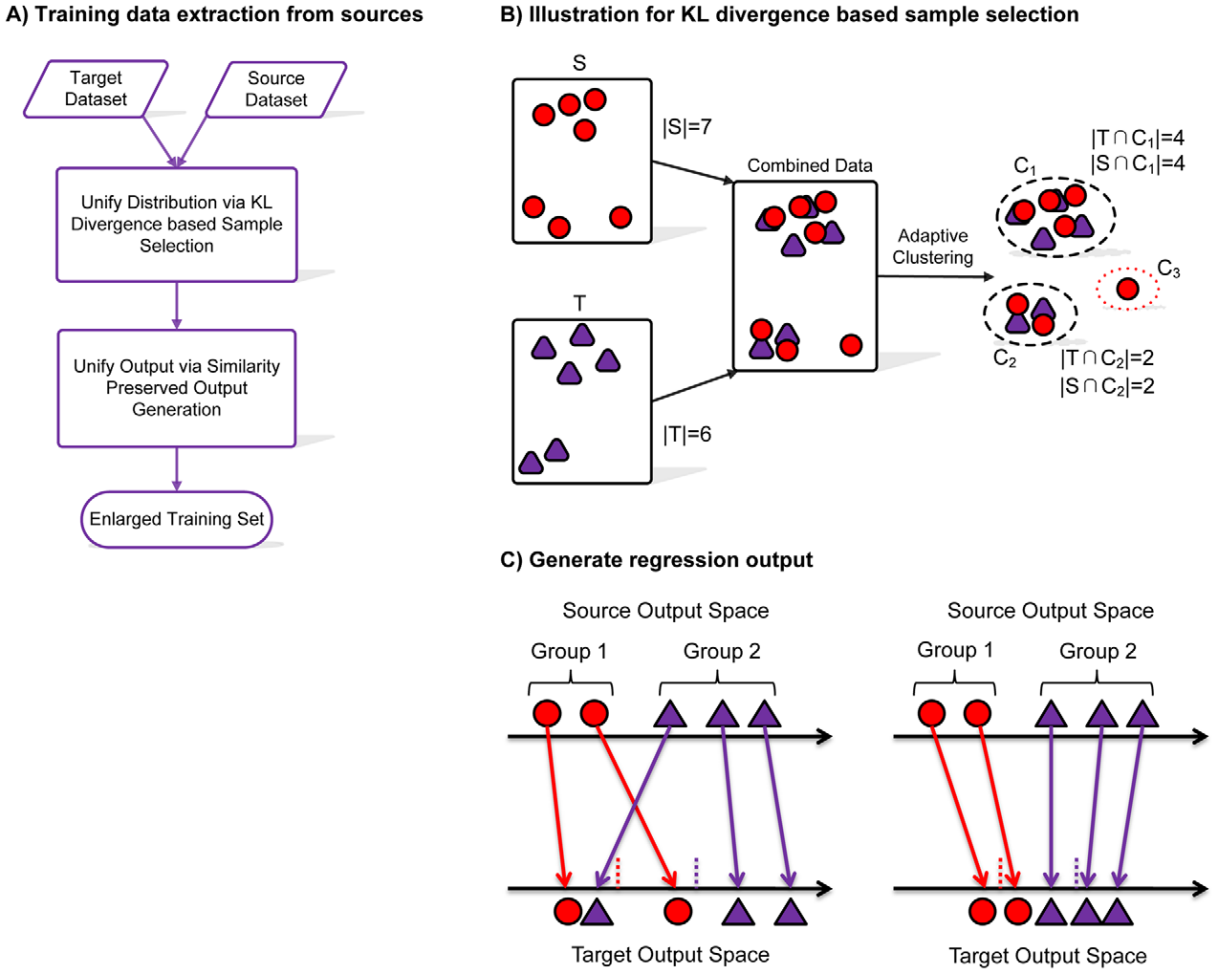
General outline of HEGS model.

Specifically, Figure 2 presents a general outline of HEGS model. The basic idea of HEGS is to select a subset of source examples similar in distribution to the target data to improve the prediction ability of the model trained from target domain; All the selected instances are “re-scaled” and assigned new output values from the labeled space of the target task. In Figure 2A, it can be seen that the training set in target domain can be enlarged based on the data transferred from source domain, by a two-steps procedures, *i.e.*, (1) data distribution unification between the source domain and target domain, and (2) output spaces unification between source domain and target domain. These two steps are described in Figure 2B and Figure 2C, where their details will be explained in the following section.

### Details of HEGS model

#### Definition and notation

In this section, we start by presenting the definitions and notations in HEGS model for target-specific siRNA efficacy modeling. In our study, the siRNA affinity data were generally split into source domain and target domain. This is naturally suitable for our 10 cross-platform datasets or the 31 sub-datasets, where one dataset can be selected as the target domain and the remains are taken as source domains. We want to investigate how the siRNA efficacy prediction model from target domain can be influenced by the knowledge transferred from the source domain, and provide novel clues on our target-specific siRNA design.

Supposing that we already selected one dataset, either from the 10 cross-platform datasets or the 31 sub-datasets, as the target dataset; then the mathematic notions used in our study are listed in the following:

The training set from the target source is denoted as 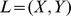, where 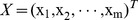 is the data array (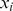 is the column feature vector) and 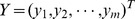 denotes the corresponding regression outputs (siRNA affinity); the test set from target set is defined as 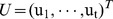 where 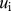 is the column feature vector data array, here we use the 497 feature representation for siRNA aforementioned [19]. The goal is thus to predict regression outputs for 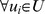 such that the predictive siRNA efficacy value is close to the true value. We take the remain 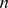 datasets as *n* tasks in source domains, denoted as 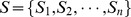, and 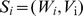 where 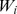 is the data array like 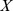 in target domain and 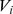 is the corresponding outputs.

It should be noted that the outputs (siRNA affinity) of the target data and the source data are allowed to be heterogeneous, which is defined as the same feature vectors with different outputs in different datasets. A traditional method to unify the heterogeneous outputs is to apply min-max normalization to transform the source outputs into the same scale as the target outputs. However, this idea requires that the source output spaces must be linear to the target output space, which is often not accordance with our problems. It has been suggested in our previous study that the normalization strategy doesn't work well for large-scale cross-platform siRNA data [20].

#### Linear ridge regression

Given the training set 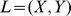 from target domain, a linear ridge regression model minimizes the following cost can be built to train a siRNA efficacy prediction model:
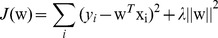
(1)where 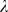 is a positive regularization parameter that controls the trade-off between the bias and variance of the estimate. The predicted label (i.e. 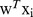) of a new unlabeled example 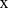 is:
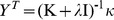
(2)where 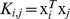 and 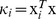, in which kernel trick can be easily applied [20].

It should be noted that we just use such a simple yet efficient liner ridge regression model to train our siRNA efficacy prediction model, since model selection is not our main focus here, and we concentrate on how to take advantage of different sources to help build the target predictive model. Considering the performance of linear regression model is comparable to most of the more complex methods on siRNA efficacy prediction, we employ the linear ridge regression to train a basic siRNA efficacy prediction model.

#### Heterogeneous regression for siRNA efficacy prediction

The heterogeneous regression (HEGS) model is based on a transfer learning paradigm initially proposed by us in machine learning community [27]. The basic algorithm of HEGS is described in Table 2. For each of the source tasks, HEGS first selects a subset of examples that is similar to the target data in distribution evaluated by the Kullback–Leibler (KL) divergence (Step 3). The algorithm then generates new outputs for each of the selected instances in Step 4. With the new training data (Step 5), the algorithm then returns a regression model in Step 7 [27]. The whole algorithm is soundly derived with a theoretical generalization bound [27]. Step 3 and 4 are the two key steps used in this model, which will be described in the next two sections respectively.

**Table 2:** The HEGS Algorithm.

Step	Procedure
	Input: Target training data 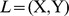 ; Target test data 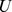 ; Source tasks 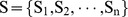
	Output: Regression model parameter 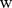
1	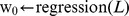 //Initial parameter
2	For each 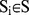 do
3	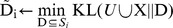 //KL divergence based sample selection
4	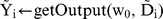 //Similarity preserved output generation
5	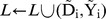
6	end
7	Return 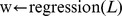

#### KL divergence based sample selection

The basic idea of KL divergence based sample selection is illustrated in Figure 2B. Intuitively, the goal of the clustering-based KL divergence is to perform clustering on the combined data set (target data and source data), and then select the source data that are similar to the target data in distribution evaluated by the clustering-based KL divergence [27] , as defined in Equation (3), where smaller KL divergence is preferred here.
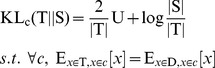
(3)Where 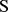 denotes the source dataset while 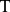 denotes the target dataset. 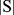 and 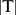 denote the data size of 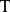 and  respectively. 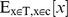 denotes the centroid of data from 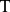 in cluster 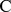, and 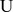 is defined as the following:
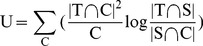
(4)Where 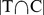 represents the number of samples in 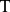 that are contained in cluster 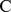, and similar for 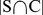. For example, in Figure 2B cluster 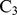 will not be selected because the sub-term in equation (3) tends to be infinity, which makes KL very large.

#### Similarity Preserving Output Generation

After selecting the samples from source data, we present the similarity preserved output generation method [27] to generate new outputs for the selected instances where their similarity in the original output space can be preserved in the target output space, as illustrated in Figure 2C. Basically it contains two steps. First, initial outputs of the source samples are generated by the regression model learnt from the initial training set, *i.e.*, 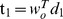, where 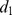 is the data sample vector and 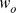 is the regression parameter learnt from the initial target training data. Next, the source samples are grouped, which can be generated from clustering on the regression values in the source output space. Then their assigned outputs are modified towards their group centers to preserve their original similarity. That is, if 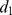 and 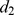 are similar in their initial output space, the new output 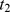 of 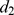 should be also similar to 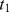 of 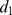. Therefore the values of 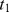 and 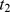 are modified to make them closer to their center [27]. In Figure 2C group centers are marked with dash lines in the target output space.

## Results

Using ten cross-platform siRNA datasets and 31 target-specific sub-datasets, a number of tests (Tests 1–6, which can be referred in File S1) were performed. Our results were also demonstrated in two parts, corresponding to the general workflow of our experiment design as illustrated in Figure 1: (1) Firstly, the regression accuracies of HEGS, SCM and the baseline (traditional linear ridge regression) on the 10 cross-platform datasets were compared (Tests 1–3), and (2) Based on the 31 sub-datasets, the target-specific siRNA efficacy analysis and siRNA feature selection at an mRNA-level were performed (Tests 5–7). In all the tests, the root mean square error (RMSE) was adopted as the performance evaluation. In addition, the paired *t*-test was conducted to verify the statistical significance between two different models.

### HEGS for cross-platform siRNA efficacy prediction

#### Comparison between the baseline strategy and a simple data combination and normalization strategy in siRNA efficacy prediction

To learn whether a simple combination and normalization method can improve the siRNA efficacy prediction or not, we compared it with a normal single platform regression model on the 10 cross-platform siRNA datasets. In this study, we randomly selected 50% of the data from each dataset as the training data to train a linear ridge regression model, and then tested it on the remaining 50% of the data. The process was repeated 10 times and the average RMSE for each dataset was calculated. This test strategy was taken as the baseline method here (Test 1). The result was compared with another test strategy (Test 2), in which the same procedure was applied except that we treated each dataset as target dataset respectively, and we randomly selected 50% of the data in target dataset to combine with all the data in other datasets (source dataset) as training data to train the model, and tested it on the remain 50% of the target dataset. From Table 3, we can see that even all the data were normalized in the same range [0,1], and the data from source datasets were add to the training data, the prediction accuracy was still not improving. Moreover, worse results in almost half of the datasets were founded. Based on this study, we draw the conclusion that simple combination and normalization of cross-platform data source provides little improvement on the siRNA efficacy prediction of a particular platform, which encourages us to apply more sophisticated HEGS to integrate data across different dataset.

**Table 3:** Comparison of the baseline strategy and a simple data combination and normalization strategy in siRNA efficacy prediction.

Test	RMSE				
	D1	D2	D3	D4	D5
Test 1	0.1556	0.2639	0.1414	0.2649	0.1766
Test 2	0.1749	0.2513	0.1610	0.2595	0.2502

	D6	D7	D8	D9	D10
Test 1	0.2704	0.3580	0.2363	0.1965	0.3105
Test 2	0.2659	0.2254	0.3357	0.1923	0.2757

#### Comparison between the baseline strategy and HEGS in siRNA efficacy prediction

**Figure 3:**
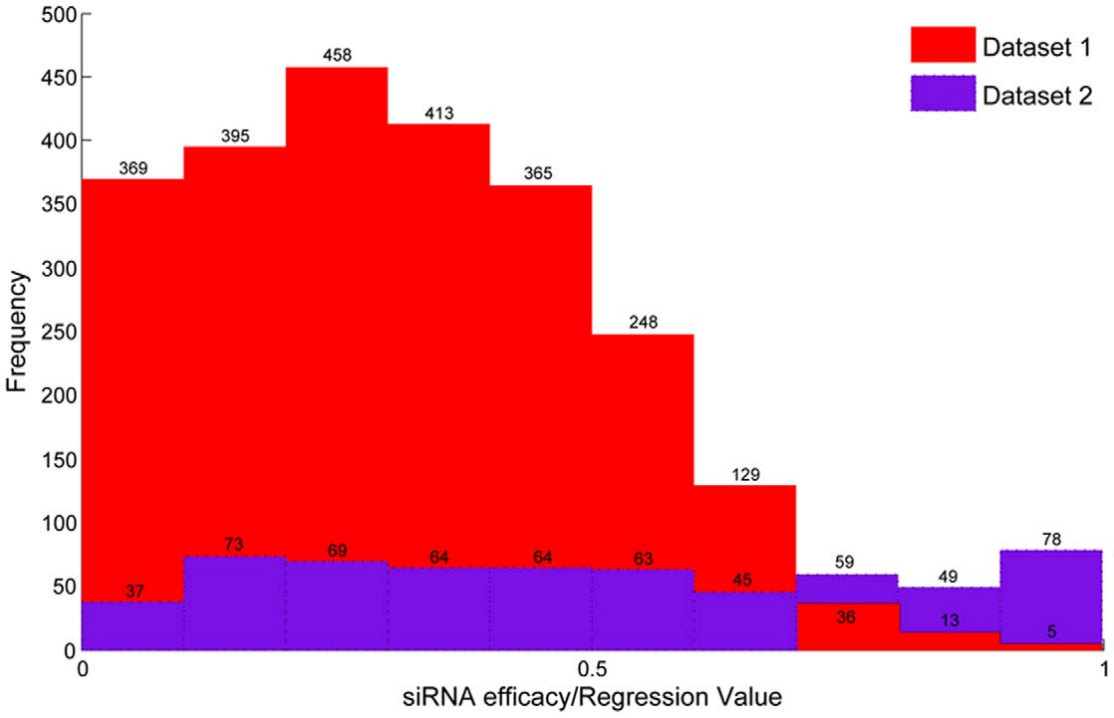
Distribution difference of the output values in Dataset 1 and 2 (Although the output values of Dataset 1 and 2 are scaled to [0, 1], the distribution between these two datasets is different).

**Figure 4:**
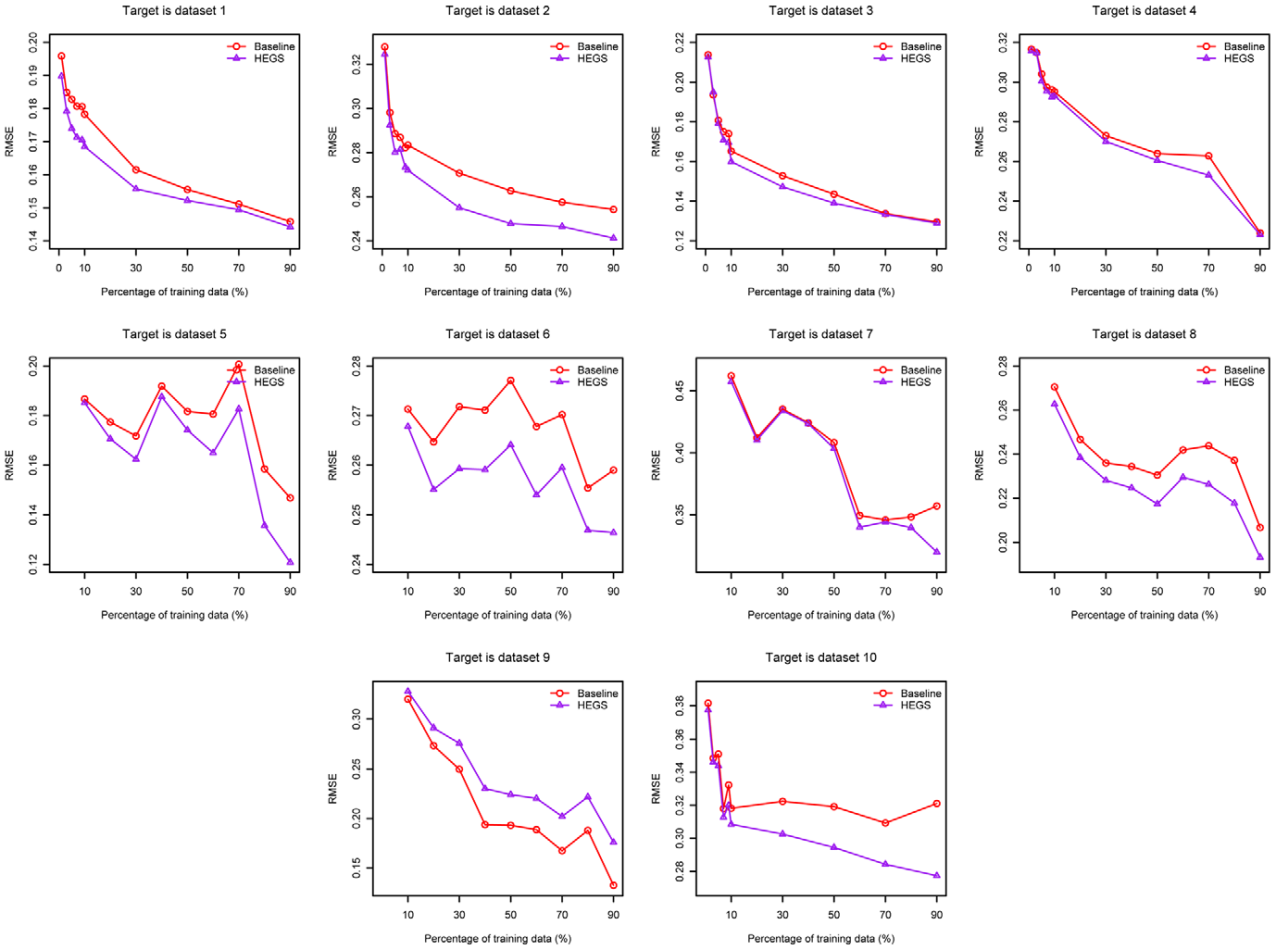
Comparison between the baseline strategy and HEGS in siRNA efficacy prediction (the parameter 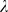 of ridge regression were kept the same).

Comparison between baseline and HEGS was performed on the 10 cross-platform datasets respectively. It should be noted that due to different bio-experimental conditions aforementioned, the outputs of different datasets are heterogeneous. As an example, Figure 3 illustrated the distributions of the output values of the first two datasets. In this study, we set each of the 10 cross-platform datasets as target task individually, while the rest datasets were taken as source tasks to train the HEGS model respectively (Test 3). It was compared with the baseline strategy trained with various different percentages of data from each target dataset, to examine the impact of the size of training set on the performance of the model. The testing results averaged on 10 runs were reported in Table 4 and Figure 4.

**Table 4:** Pair *t*-test *p*-values for the comparison between HEGS and baseline with different percentages of training data for 10 datasets.

Pair *t*-test on baseline and HEGS					
Training data percentage	10%	30%	50%	70%	90%
*p*-value	0.00087	0.0022	0.0032	0.0056	0.014

From Figure 4, it can be clearly seen that HEGS achieved better performance as compared to baseline under various training data percentages for nearly all the experiments, except for dataset 9, probable due to its relatively small data samples. Pair *t*-test evaluation showed that HEGS is significantly superior to baseline in siRNA efficacy prediction with different percentages of training samples (Table 4), which indicated that the knowledge transferred from source domain do help for siRNA efficacy modeling in the target domain. Moreover, as shown by the *p*-values, the significance of the difference between two methods was negatively correlated with the percentage of the training data, suggesting that our HEGS can greatly improve the accuracy of the siRNA affinity modeling especially when the existed RNAi data is insufficient.

### HEGS for target-specific siRNA efficacy analysis

#### Target-specific siRNA efficacy modeling

**Figure 5:**
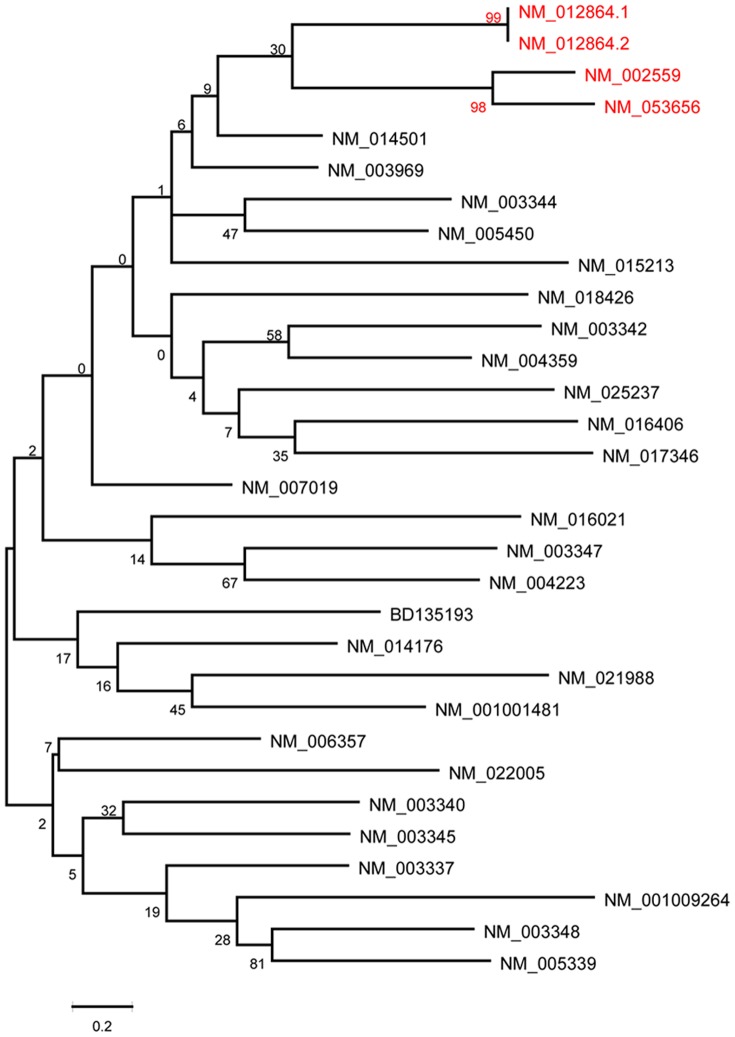
Discover the target genes with high homology using the phylogenetic tree (Three couples of homologous genes were identified: NM_002559 and NM_053656, NM_005339 and NM_003348, NM_012864a and NM_012864b).

In this study, we grouped the siRNAs binding to 31 mRNAs in Novartis's dataset [17] as 31 sub-datasets, to investigate the target-specific siRNA efficacy modeling under various *in silico* strategies. We first ran nucleotide BLAST on the 31 mRNAs to find whether there exist any two genes with highly homology, which resulted in two couples: (1) NM_002559 and NM_053656, two homologous genes with a max identity referring to 95%; and (2) NM_012864.1 and NM_012864.2, which are two different versions of the same gene. Then nucleotide sequences of the 31 target mRNAs were downloaded from Genbank and a phylogenetic tree was built using MEGA 5.03 [28] to find the genes with highly genetic relation, as showing in Figure 5. It can be seen that the same two clades of the tree were also identified with bootstrap values larger than 90, indicated that the genes on these two clades are genetically related to each other (Highlighted in red in Figure 5). The results of ClustalW alignment of these two clades are provided in Figure S1 and Figure S2. For each clade, the sub-dataset corresponding to one of the genes was taken as target task and the other one was taken as source task to train the HEGS, respectively (Test 4). This test was particularly designed to address the first problem as we proposed in INTRODUCTION: when we design siRNAs target on a specific mRNA, but lack of its affinity data towards this target, can we leverage the existed siRNA affinity information targeted on its highly homologue genes? In addition, in order to further investigate the influence of source data on the target data, as the second problem we proposed in INTRODUCTION, we want to examine whether it is better to transfer from the most similar tasks (homologue genes) than those from all the source tasks (all the genes) for siRNA design? With these two questions in hand, we compared Test 4 with another two test scenarios: (1) HEGS with all the remain data samples in Novartis's as the sources task (Test 5), and (2) the baseline model without transfer (Test 6).

**Figure 6:**
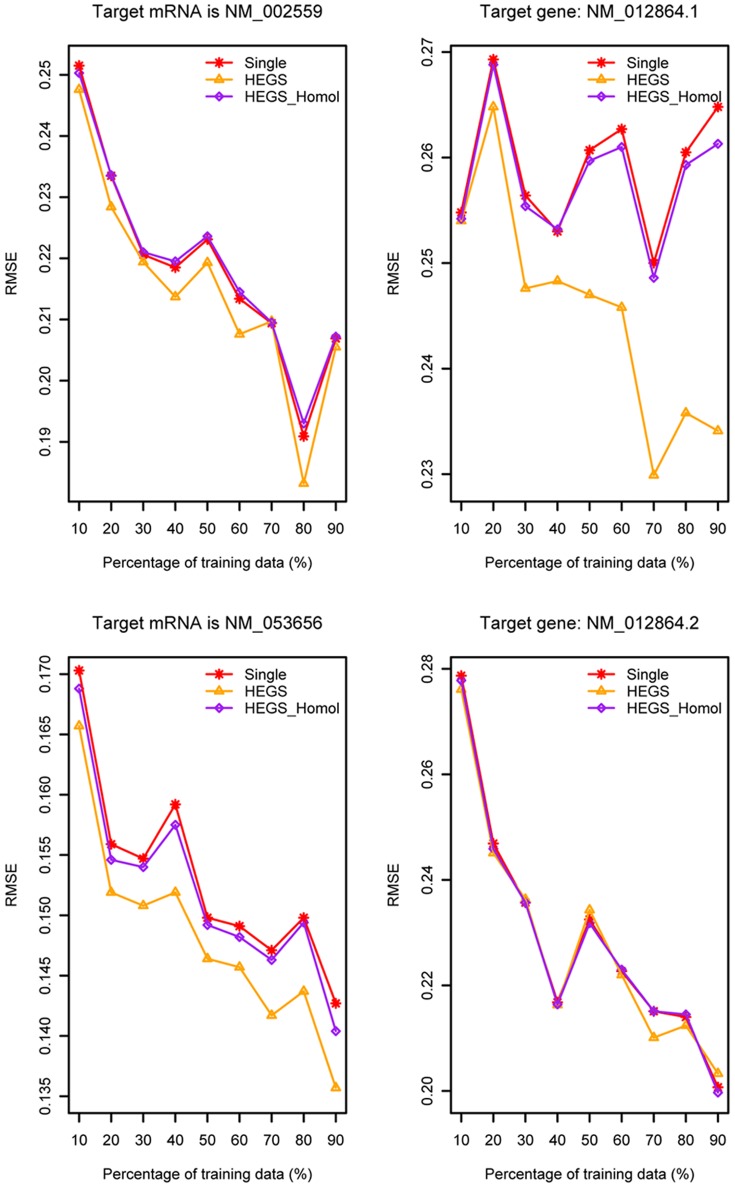
Target-specific siRNA efficacy modeling with different test strategies (Red line represents the RMSE obtained by the baseline strategy, labeled as “Single”. Yellow line represents the RMSE obtained by transfer from the data of all the other genes, labeled as “HEGS”. Blue line represents the RMSE obtained by transfer from the data of the homologues gene, labeled as “HEGS_Homol”).

This study was performed on 2 clades of the tree, totally containing 4 sub-datasets. Each dataset was selected as the target task respectively, and compared in three different test sceneries (Tests 4–6). The test data in the target task was hold with 10% to 90% of the whole data. The results on 10 runs of the three tests for the 4 mRNAs were plotted in Figure 6, from which we observed that compared with the baseline, extracting samples from highly homolog genes indeed help improve the learning accuracy, especially when the training dataset is small. Furthermore, with a little surprising, samples extracted from all the other genes help to achieve more significant improvement, as the performance of Test 5 outperformed the other two in almost all cases with different percentages of training data in the target domain, especially for the two sub-datasets corresponding to genes “NM_053656” and “NM_002559”.

**Figure 7:**
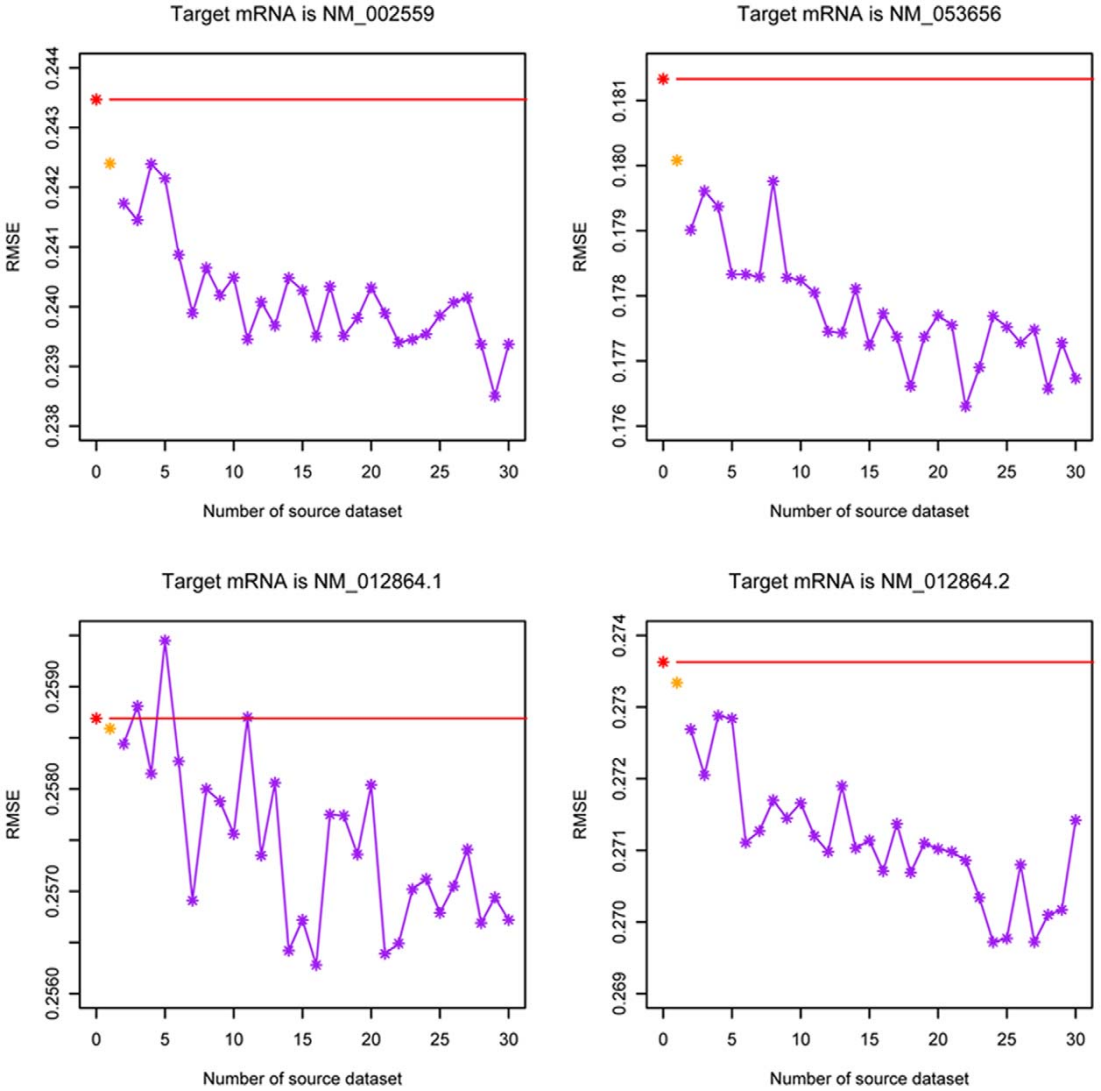
Target-specific siRNA efficacy modeling with different amount of source data (Red node represents the RMSE value obtained without transfer. Yellow node represents the RMSE value obtained by homologue gene transfer. While other blue nodes represent the RMSE values obtained by randomly added the 31 sub-dataset one by one to enlarge the source domain in transfer).

Does this means that the more number of data it contained in source domain, the better improvement it obtained by knowledge transfer for target-specific siRNA affinity modeling? We found that this is not always the case when we examined the amount of source data in a following way (Test 7): we randomly added the 31 sub-dataset one by one to the source domain to enlarge it, and compared it with the baseline and HEGS_Homol at every step, and this procedure was repeated for 10 times. The average results were illustrated in Figure 7. We found that the results are fairly random for all the 4 mRNAs. Although knowledge transfer always achieved a better result compared to that of baseline, it is not necessarily that the more number of source domain in transfer performed better than that of less one. This test indicated, at least for the current dataset, there exists no significant positive correlation between the data amount of source domain and the performance of target-specific siRNA affinity modeling. From the biological point of view, we still recommend to select the most homologue genes as source task to transfer for the target-specific siRNA design, especially when large number of siRNA affinity data for this source mRNAs existed. Such transfer will be “safer” since the source mRNA and target mRNA are evolutionarily closed.

#### Feature selection for target-specific siRNA design

In this section, we further studied how the domain transfer can be helpful for target-specific feature selection for siRNA design. As an example, we selected one couple of genes (NM_002559 and NM_053656) on the two homologue clades in Figure 5, since they are highly homologous. It should be noted that we can similarly check other homologue pairs here, which will not be discussed in detail. The basic idea of this study is that we want to examine whether HEGS-based feature selection on the siRNA data for one specific mRNA can be helpful for the siRNA design for another mRNA, provided that these two mRNAs are homologue. In this study, the siRNA data binding to mRNA NM_002559 were used as an independent test dataset. Its homologue gene, Gene NM_053656 with its binding siRNA was set as a target dataset and the remaining siRNA in Novartis's dataset was taken as source dataset. We want to mimic such a scenario in in-silico siRNA design: if we want to design efficient and specific siRNAs for the gene NM_002559, while there doesn't exist any prior knowledge or experimental siRNA binding affinity data to this gene, can we use its homologue gene NM_053656 to help its siRNA design, provided that there existed a few but not sufficient siRNA affinity data to NM_053656? We proposed that we can use HEGS to improve the feature selection for target NM_053656, and then these selected features after HEGS can be used for improved siRNA design for target NM_002559.

Firstly before HEGS, we used two criteria proposed in the previous study [10] to select siRNA features which were related to the siRNA efficacy for NM_053656 on the current dataset: (1) the selected features must have a correlation of at least 0.1 with its siRNA efficiency, and (2) this correlation is statistically significant at 0.05 level to distinguish active and non-active siRNA. As a result, 28 features were identified with a significant correlation to siRNA efficacy. The features ranked with their correlation coefficient were listed in order in Table 5.

**Table 5:** Selected features for siRNA targeted on NM_053656 before HEGS.

Feat. ID	Feat No	Features	siRNA for NM_053656		siRNA for NM_002559	
			R	*p*-value	R	*p*-value
1	477	dG(NT[1,2]) - dG(NT[18,19])	0.5248	6.77e-03	0.4936	5.49e-05
2	415	dG in NT[1,2]	0.5151	3.95e-05	0.4359	1.65e-04
3	41	U @ NT1	0.4189	3.99e-03	0.4337	1.78e-05
4	498	dG(NT[1,2,3,4]) – dG(NT[18,19])	0.3815	0.033	0.2986	0.011
5	22	G @ NT1	−0.3634	0.007	−0.3975	1.02e-04
6	344	UCUG in NT[1..19]	0.3499	8.0e-05		
7	274	GCAC in NT[1..19]	0.3278	0.046		
8	365	CACU in NT[1..19]	0.2727	0.0043		
9	469	NT18 forms bond	−0.2689	0.0089		
10	364	CACG in NT[1..19]	0.2597	0.0496		
11	468	NT17 forms bond	−0.2511	0.0059		
12	315	UGCA in NT[1..19]	0.2497	4.3e-04		
13	281	GCUU in NT[1..19]	0.2480	1.97e-05		
14	155	CCA in NT[1..19]	−0.2478	0		
15	115	GGA in NT[1..19]	−0.2449	0.0084		
16	339	UCGA in NT[1..19]	0.2427	0.0134		
17	333	UUCU in NT[1..19]	0.2402	0.0074		
18	329	UUUU in NT[1..19]	0.2153	0.0045		
19	94	CC in NT[1..19]	−0.1998	5.35e-04		
20	495	GC content <0.55	0.1911	0.013	0.2658	0.0054
21	434	dG in NT[1..4]	0.1713	0.025	0.3531	0.0015
22	138	UUC in NT[1..19]	0.1591	0.025		
23	108	ACG in NT[1..19]	0.1488	0.023		
24	2	GC content in NT[1..19]	−0.1457	3.44e-03	−0.3389	0.0051
25	42	U @ NT2	0.1345	0.0248		
26	57	U @ NT17	−0.1324	0.0209		
27	433	SUM of dG	0.1297	8.24e-03	0.3388	0.0126
28	126	GCC in NT[1..19]	−0.1254	0.0025		

Then we transferred knowledge from all the other mRNA dataset by HEGS to help to improve the size of target siRNA dataset on NM_053656, and used this enlarged dataset to select the features under the same criteria. The transfer process was repeated 1000 times and the top-28 features were selected and ranked with their frequency of occurrence, as shown in Table 6.

**Table 6:** Selected features for siRNA targeted on NM_053656 after HEGS.

Feat. ID	Feat No	Features	siRNA for NM_053656	siRNA for NM_002559	
			R	*p*-value	t-test *p*
**1**	**494**	**‘GC content <0.55’**	**+**	**0.2658**	**0.0054**
**2**	**485**	**‘GC content >0.45’**	**+**	**−0.2428**	**0.0019**
3	493	‘GC content <0.6’	+		
**4**	**476**	**dG(NT[1,2]) - dG(NT[18,19])’**	**+**	**0.4936**	**5.94E-05**
5	484	‘GC content >0.4’	+		
**6**	**414**	**‘dG in NT[1,2]’**	**+**	**0.4359**	**0.0001**
**7**	**497**	**dG(NT[1,2]) – dG(NT[16,17,18,19])’**	**+**	**0.2986**	**0.0113**
8	492	‘GC content <0.65’	+		
9	483	‘GC content >0.35’	+		
10	80	‘AU in NT[1..19]’	−		
11	86	‘UA in NT[1..19]’	+		
12	491	‘GC content <0.7’	+		
**13**	**432**	**‘SUM of dG’**	**+**	**0.3389**	**0.0126**
**14**	**1**	**‘GC content in NT[1..19]’**	**−**	**−0.3388**	**0.0051**
15	96	‘AAU in NT[1..19]’	−		
16	85	‘GC in NT[1..19]’	+		
17	104	‘AUU in NT[1..19]’	−		
**18**	**449**	**‘SUM of dG4’**	**+**	**0.3491**	**0.0118**
**19**	**91**	**‘CG in NT[1..19]’**	**+**	**−0.3070**	**0.0099**
20	126	‘UAA in NT[1..19]’	−		
21	490	‘GC content <0.75’	−		
22	496	dG(NT[1,2,3,4]) – dG(NT[18,19])’	+		
23	495	dG(NT[1,2,3,4]) – dG(NT[16,17,18,19])’	+		
24	115	‘GGG in NT[1..19]’	−		
25	471	‘G stretch of length > = 3’	−		
**26**	**450**	**‘Folding in NT[1..19]’**	**−**	**−0.3869**	**0.0011**
27	136	‘UUU in NT[1..19]’	−		
28	102	‘AUA in NT[1..19]’	−		

Compared from Table 5 and Table 6, we can see that there indeed exists a considerable difference between the selected features before and after HEGS. The probable reason underlying is that currently the accumulated siRNA affinity data for a specific mRNA target (NM_053656) may not be sufficient, thus the feature selection performed on the insufficient dataset may not reveal the truly significant features for target-specific siRNA design. With the more experimental siRNA data accumulated, more robust and accurate feature selection can be achieved. Furthermore, in the next section we will see these features are better in modeling siRNA affinity for the independent test target NM_002559.

#### Validation of the selected features

To validate whether the HEGS-based selected features were superior to target-specific siRNA design, regression models were trained with the two feature lists for siRNA efficacy prediction on the independent test dataset NM_002559, respectively. Traditional linear ridge regression was applied to construct the predictive model. Different percentages of training data and 10 times 10-fold cross-validation were applied to evaluate model generalization. Results were given in Table 7. It was clear that the predictive model using HEGS-based features slightly outperformed that using the features without knowledge transfer.

**Table 7:** Comparison of RMSE for the prediction model on the target NM_002559 with HEGS-based features and features without transfer, under different training data percentages.

NM_002559	0.1	0.3	0.5	0.7	0.9	10-fold CV
Without transfer	0.2787	0.2480	0.2370	0.2327	0.2234	0.2221
Transfer	0.2484	0.2332	0.2234	0.2220	0.2077	0.2095

In addition, we investigated the relations between the HEGS-based 28 features and test siRNAs. The bold items in Table 6 represent newly identified features with statistically significances to distinguish active and non-active siRNA targeted on NM_002559. These features are derived based on HEGS for target NM_053656, which is a homologue gene of NM_002559. All the top 7 features belonging to the categories of thermodynamic features and compositional features (GC contents), are statistically significant for siRNA design on NM_002559. The motif features and structure features which were important for siRNA design on NM_053636, however, contributed much less to that on NM_002559. The *p*-values of these features were either between 0.05∼0.1 or larger than 0.1 (not shown as blanked in Table 5 and Table 6). Details of these newly identified features will be discussed in the next section.

## Discussion

### HEGS can improve learning accuracy for siRNA efficacy prediction

In our study, the results of our *in silico* tests demonstrated that when the number of target siRNA data is limited, HEGS can improve the prediction for siRNA efficacy by increasing the potential source of training data. Compared to the simple combination of different datasets, HEGS provides an effect way to solve the problem brought by the heterogeneity of different datasets. Due to the different data distributions of the source data, direct use of the source examples is unsuitable to the learning with limited improved predictive accuracy. It should be noted that the “negative transfer” should also be avoided as it can negatively affect the learning results. Like the experiment 9 in Test 3, sometimes redundant source data were harmful for model construction [29].

### Design of siRNA based on the specificity of target mRNA

It has been confirmed that the system-level factors of mRNA, like the target mRNA cellular abundance and turnover rate, influence siRNA efficacy [24], [25]. We also found in our previous study that in siRNA efficacy prediction, there indeed exist certain efficacy distribution diversity across the siRNAs binding to different mRNAs, and this distribution diversity seems to be weak within the siRNAs binding to the same mRNAs [20], [21]. These findings help validate the observation that the properties of mRNA do have impact on the efficiency and specificity of siRNA design, since certain data heterogeneity has been detected across the siRNAs binding to different mRNAs. Therefore we hold the reason to improve the target-specific design of siRNA from the viewpoint of considering the heterogeneity across siRNAs binding to different targets.

In Test 4∼6, we studied whether HEGS can help train an accurate predictive model given that the siRNA data towards a specific target mRNA is insufficient. In reality, as we often have no prior knowledge about the siRNAs binding to the target gene, we focus on the siRNA data available with their target gene that is the homology to the gene to silence. We no longer adopt the same 497 siRNA features described in the literature [19] to construct the predictive model since such a feature space is too large for accurate and time-saving analysis. The similar characteristics within the homologous genes inspired us to discover some features that are significant to siRNAs binding to a specific target mRNA. From the results of Table 5 and Table 6, we confirmed that the HEGS-based features selected from the siRNAs binding to homologous gene NM_053656 do enhance the predictive accuracy of siRNAs binding to gene NM_002559. Further analysis on the selected features allowed us to identify that some of these features (presented in bold) after HEGS are significant as well for the siRNAs binding to NM_002559. It can be observed from Table 5 that all the significant features belong to the categories of compositional features and thermodynamic features. In the following we just discussed these features that are important for target-specific siRNA design.

#### Compositional features

The connection between GC content and siRNA efficacy is focused by a few research groups [8], [12], [19]–[21]. However, the association between GC content and siRNA functionality is described differently among studies. For instance, Klingelhoefer et al. [19] suggested that siRNA candidate sequences with GC content in the range of 35–73% have an increased potency. Matveeva et al. [18] identified a GC content of 20–53% as more advantageous for siRNA design. Table 5 revealed three features of GC content as significant for predicting siRNA efficacy in the independent test dataset: GC content <55% (Feat.ID = 1), GC content >45% (Feat.ID = 2) and GC content in 19 nt antisense strand (Feat.ID = 14). The positive correlation of feature GC content < 55% shows that siRNA sequences with these features have an increased efficacy to affect the target mRNA NM_053656 or NM_002559, which is consistent with the upper limit for GC content in Matveeva's literature. Similarly, the negative correlation of feature GC content in 19 nt antisense strand indicates that very high GC content may be a negative determinant of functionality since it can inhibit the dissociation of the siRNA duplex, which is necessary for RISC loading. The lower limit for GC content can be discovered from the feature GC content >45% (Feat.ID = 2), which is, however, negatively correlated with siRNA efficacy in test dataset. Hence, we examined 73 siRNAs of which the GC content are larger than 0.45 in the test set and found only 16 of these siRNAs have a high efficacy (efficient value <0.3). Moreover, all the 16 efficient siRNAs share a GC content between 0.45 and 0.55, which corresponds with the other two features. Previous studies argued that very low GC content is associated with decreased functionality, presumably due to lowered target affinity and specificity [22]. Such inconsistence, as well as the variety of the percentage of GC content between studies, may result from the different characteristics of target mRNAs as well as the improperly using of different combined siRNA data in previous studies.

#### Thermodynamic features

Three of the top 7 features after HEGS are thermodynamic features, indicating the critical role of the thermodynamic properties of siRNA in duplex unwinding and strand retention by the complex. We found that the thermodynamic stability difference between the 5′ and 3′-end of the antisense strand (Feat.ID = 4) is more important than the separate 5′ features (Feat.ID = 6), which has been pointed out by several studies [12], [18]. Moreover, such difference between the first and last dinucleotide (dG1-2 – dG18-19, Feat.ID: 4) seems to be more notable than the other combinations of the end strand stability terms, such as dG1-4 – dG18-19 (Feat.ID = 22) and dG1-4 – dG16-19 (Feat.ID = 23), which accords with the results that the first tetranucleotide (dG1-2, Feat.ID = 2) is more critical than the first dinucleotide (dG1-4, Feat.ID = 21). It is typical of the efficient siRNA duplexes to contain less stable 5′ ends and more stable 3′ end of the antisense strand. In addition, the total free energy (SUM of dG, Feat.ID = 13 and SUM of dG4, Feat.ID = 18) was also included in the significant features, which implies that the stability of longer stretches of neighboring nucleotides may have an impact on siRNA efficacy as well.

#### Structural features

A negative correlation between the self-folding (Feat ID: 26) of a siRNA and its silencing potential is identified, which emphasizes the probability that the presence of siRNA secondary structure may affect the interaction between the guide RNA and its target.

The remaining features are not important to all the siRNAs. In particular, the motif features selected from the siRNA data binding to NM_053656 are less significant to the siRNA binding to NM_002559. Thus we speculated that these features won't guarantee the high efficacy of siRNAs undoubtedly, hence they should not be used to improve the design of target-specific siRNAs.

Throughout the features selected in our study, they are somehow distinct from the current generally acknowledged siRNA design rules in the field of RNAi. We cannot simply make the judgment on whether a certain feature is important or not by simply statistical evaluation. However, from our study we confirmed that the joint siRNA design rules should be considered along with the target-specific design rules to improve the efficiency of siRNAs to reduce their OTEs for a specific target.

With the increasing need of therapeutic siRNA, it requires us to develop the siRNA drugs that can silence disease genes effectively and safely. This can be analogues to the Quantitative Structure Activity Relationship (QSAR) study in chemistry community [30], where the molecules binding to one target should have a specific QSAR model for biological activity prediction rather than mix all the targets together. From this point of view, we consider that the siRNAs binding to one target mRNA may also contain inherent characteristic we may not uncover, and should hold their own efficacy predictive model. For a particular target gene, this can be achieved by firstly design the siRNAs targeting the homologous genes in animals such as mouse to derive the specific design rules, and then apply such domain transfer based design guidelines to the in-vivo siRNA design in human clinical trials.

## Supporting Information

Figure S1 **Alignment result of genes NM_002559 and NM_053656.**
(TIF)

Figure S2 **Alignment result of genes NM_012864.1 and NM_012864.2.**
(TIF)

File S1 **A detailed description of each test performed in our study.**
(DOCX)
