# Supplementary figures and images for: In Silico Target-Specific siRNA Design Based on Domain Transfer in Heterogeneous Data
Source: PLoS One. 2012 Dec 21;7(12):e50697. doi: 10.1371/journal.pone.0050697 (PMC3528743; doi:10.1371/journal.pone.0050697)

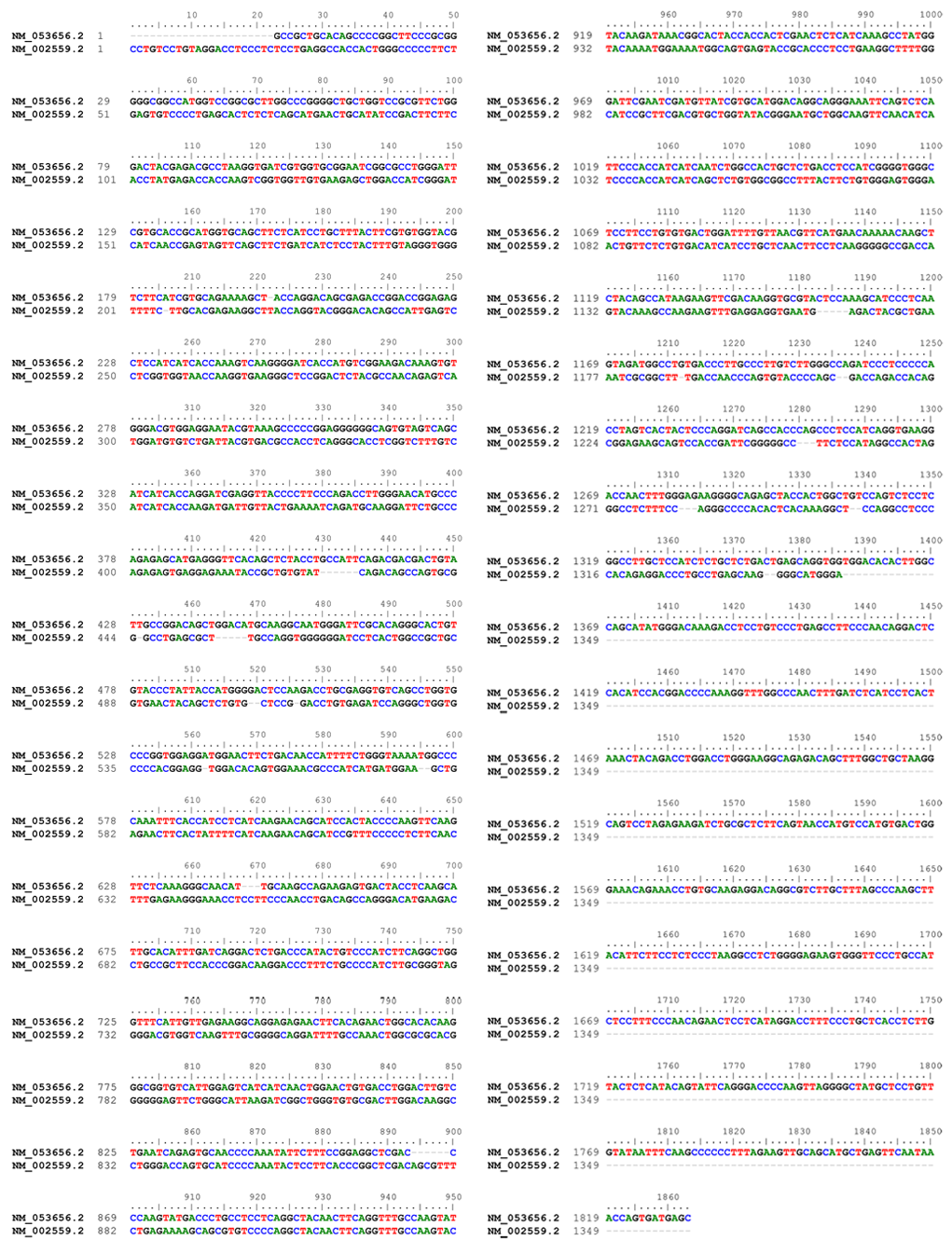

Supplement: Figure S1 — Alignment result of genes NM_002559 and NM_053656. (TIF) [file pone.0050697.s001.tif]

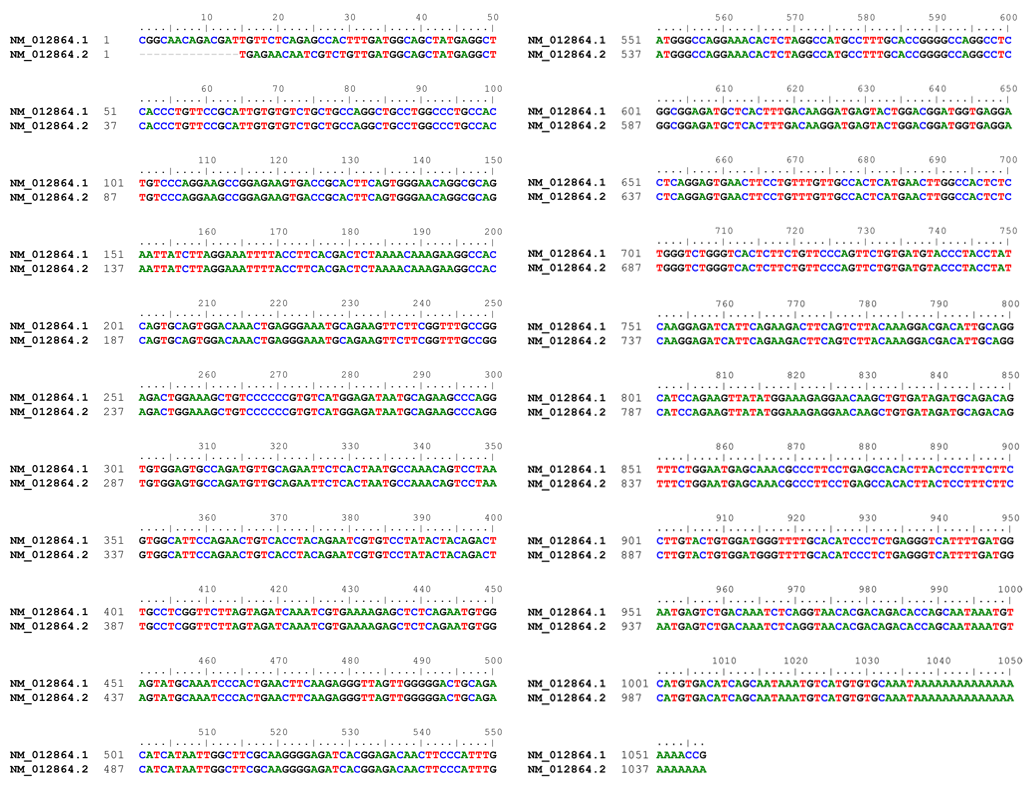

Supplement: Figure S2 — Alignment result of genes NM_012864.1 and NM_012864.2. (TIF) [file pone.0050697.s002.tif]
