# Supplementary material for: In Silico Target-Specific siRNA Design Based on Domain Transfer in Heterogeneous Data
Source: PLoS One. 2012 Dec 21;7(12):e50697. doi: 10.1371/journal.pone.0050697 (PMC3528743; doi:10.1371/journal.pone.0050697)
Supplement: File S1 — A detailed description of each test performed in our study. (DOCX) [file pone.0050697.s003.docx]

## Descriptions of each test performed in our study

Test 1: we randomly selected 50% of the data from 10 cross-platform datasets as the training data to train a linear ridge regression model, and then tested it on the remaining 50% of the data respectively. The process was repeated 10 times and the average RMSE for each dataset was calculated. This test strategy was taken as the baseline method.

Test 2: we randomly selected 50% of the data from 10 cross-platform datasets as the target dataset respectively, and the remain 50% of the data is combined with all the data in other datasets (source dataset) as training data to train the liner regression model respectively.

Test 3: we set each of the 10 cross-platform datasets as target task individually, while the rest datasets were taken as source tasks to train the HEGS model respectively.

Test 4: For each homologue gene pair in the 31 sub-datasets, the sub-dataset corresponding to one of the genes was taken as target task and the other one was taken as source task to train the HEGS, respectively.

Test 5: For each homologue gene pair in the 31 sub-datasets, the sub-dataset corresponding to one of the genes was taken as target task, while all the remain data samples in Novartis’s were taken as sources task to train the HEGS, respectively.

Test 6: For each homologue gene pair in the 31 sub-datasets, we randomly selected 50% of the data as the training data to train a linear ridge regression model, and then tested it on the remaining 50% of the data respectively.
